# Effect of baseline fluid localization on visual acuity and prognosis in type 1 macular neovascularization treated with anti-VEGF

**DOI:** 10.1038/s41433-024-03256-1

**Published:** 2024-07-31

**Authors:** Etienne Gadiollet, Laurent Kodjikian, Fanélie Vasson, Kenny Kodaday, Nicolas Chirpaz, Benjamin Wolff, Flore De Bats, Audrey Feldman, Pierre Pradat, Pierre Gascon, Thibaud Mathis

**Affiliations:** 1 Service d’Ophtalmologie, Hôpital Universitaire de la Croix-Rousse, Hospices Civils de Lyon, Lyon, France; 2 UMR-CNRS 5510 Matéis, Université Claude Bernard Lyon 1, Villeurbanne, France; 3 Centre de Recherche Clinique, Hôpital Universitaire de la Croix-Rousse, Hospices Civils de Lyon, Lyon, France; 4 Centre Ophtalmologique Maison Rouge, Strasbourg, France; 5 Pôle Vision, Clinique du Val d’Ouest, Ecully, France; 6 Centre Ophtalmologique LEO, Hôpital Privé de l’Est Lyonnais, Saint-Priest, France; 7 Département d’Ophtalmologie, Université d’Aix-Marseille, Hôpital Nord, Marseille, France; 8 Centre Monticelli Paradis, Marseille, France; 9 Groupe Almaviva Santé, Clinique Juge, Marseille, France

**Keywords:** Macular degeneration, Prognostic markers

## Abstract

**Purpose:**

To assess the prognostic value of subretinal (SRF) and intraretinal fluid (IRF) localizations in type 1 macular neovascularization (MNV) due to age-related macular degeneration (AMD).

**Subjects:**

Eyes were prospectively treated with anti-vascular epithelial growth factor (anti-VEGF) intravitreal injections (IVT) according to a Pro-Re-Nata (PRN) or Treat and Extend (TAE) regimen during 24 months. A total of 211 eyes with treatment-naïve type 1 MNV secondary to AMD were consecutively included. Eyes were divided between 2 groups according to the fluid localization: presence of SRF alone (SRF group), or presence of IRF associated or not with SRF (IRF ± SRF group).

**Results:**

At baseline the mean BCVA was 66.2 letters. SRF was present in 94.8% of eyes, IRF in 30.8%, and both in 25.6%. Data were available for 201 eyes at 12 months, and 157 eyes at 24 months. The presence of IRF at baseline was associated with lower baseline BCVA and significantly lower BCVA at 12 months (p < 0.001) and 24 months (p < 0.001). Eyes with SRF alone displayed better visual outcomes (BCVA at month 12, SRF = 74.3 letters, IRF ± SRF = 56.9 letters). In the presence of baseline IRF, fibrosis (p = 0.03) and atrophy (p < 0.001) were more frequently found at 24 months. In a multivariate model, the presence of baseline IRF was significantly associated with lower BCVA at month 12 but not at month 24.

**Conclusion:**

In type 1 MNV, the presence of baseline IRF was associated with worse visual outcomes compared to SRF alone, and more frequent atrophy and fibrosis.

## Introduction

Age-related macular degeneration (AMD) is a frequent and chronic retinal disease, associated with a significant risk of severe vision loss and a major cost for healthcare systems [1]. Macular neovascularization (MNV) defines the neovascular form of the disease (nAMD), which can lead to dramatic vision loss within a short period of time in case of exudation [2, 3]. Since the advent of anti-vascular endothelial growth factor antibodies (anti-VEGF), vision loss is generally limited, and treated patients conserve or even gain in visual acuity [2, 4]. Anti-VEGF acts by reducing vascular leak as a result of the abnormal vessels which lead to retinal dysfunction and visual loss if untreated [5]. To reduce vascular leak, several treatment protocols have been evaluated and are currently used in routine patient care. In these protocols, disease activity is assessed based upon visual acuity decline, but also (and mostly) on the presence of retinal fluid revealed by optical coherence tomography (OCT) [6, 7]. The latter is a quasi-histologic in vivo examination of the macula allowing a non-invasive assessment of the presence of fluid in the retina. The development of this technology has enabled physicians to localize the MNV-related fluid in specific retinal layers, and two main fluid compartments have been described using spectral domain (SD)-OCT: subretinal fluid (SRF) and intraretinal fluid (IRF) [8]. Many studies have since investigated the prognosis of each fluid on visual acuity. Post-hoc analyses of the CATT [9] and VIEW [10] studies first suggested that the presence of SRF at baseline was associated with favorable functional outcomes, contrary to IRF that led to worse vision. Moreover, the FLUID study [11] confirmed that an SRF-tolerant regimen was non-inferior in terms of visual acuity gain compared to a non-tolerant regimen. However, none of these studies considered the subtypes of MNV, which could nonetheless have a key impact on the fluid prognosis. The recent and consensual classification of MNV defined 3 subtypes: type 1 and type 2 MNV begin in the choroidal compartment, type 2 MNV passes through the retinal pigmentary epithelium (RPE) layer; conversely, type 3 MNV develops intraretinally and towards the outer retina layers to finally breaks through the RPE layer [8]. Recently, the FLIP-3 study found that the presence of IRF at baseline was associated with better visual outcomes than the presence of SRF at baseline for type 3 MNV [12]. The authors suggested that the pathophysiology of type 3 could explain this different prognosis, as type 3 MNV begins in the inner retinal layers (with more frequent IRF) and secondarily progresses deeper in the retina to finally mature under the RPE layer (and at this time associating with SRF) [13].

Unlike type 3 MNV, type 1 MNV develops beneath the RPE layer from the choriocapillaris, which may explain the predominance of SRF at baseline [8, 14]. It is supposed that a less frequent external limiting membrane (ELM) disruption limits fluid movement from the subretinal to the intraretinal compartment, resulting in a lower frequency of IRF [14]. As type 1 MNV is the most frequent neovascular type in AMD, we presume that the results reported by original studies, pooling all MNV subtypes and regarding the influence of the fluid localization on the prognosis, may actually be attributable mostly to type 1 MNV, the most frequent type of nAMD seen. The literature lacks supportive evidence on the prognostic impact of fluid localization in type 1 MNV [15]. We therefore conducted a study to explore the functional outcomes and prognosis of SRF and IRF on visual acuity in patients treated with anti-VEGF agent for type 1 MNV in the context of AMD.

## Methods

### Study population

This study retrospectively included consecutive eyes from 7 French retina centers between April 2014 and January 2022 treated with anti-VEGF for active type 1 MNV secondary to AMD. Inclusion criteria were: age ≥50 years, having a type 1 MNV secondary to AMD, being AMD-treatment naïve at the time of the diagnosis, and being treated with intravitreal ranibizumab (0.5 mg in 0.05 mL of Lucentis; Genentech, Inc., San Francisco, CA, US) or aflibercept (2.0 mg in 0.05 mL of Eylea; Regeneron, Tarrytown, NY, US) after a loading dose of 3 intravitreal injections during the first 3 months following diagnosis. The choice of the initial anti-VEGF drug was made at the discretion of the attending physician. Both eyes could be included in case of bilateral involvement. Exclusion criteria were: presence of polypoidal choroidal vasculopathy (PCV), having a type 2, 3, or mixed MNV, presenting a medical history of confounding retinal and macular disease (diabetic retinopathy, degenerative myopia, retinal detachment, macular hole, or any other pathology that could affect the macula), having a MNV secondary to another maculopathy (degenerative myopia, angioid streaks, inflammatory maculopathy), being concomitantly treated with intravitreal steroids or photodynamic therapy, and not being able to undergo SD-OCT follow-up or visual acuity measurement. The informed consent of patients was obtained, and a local ethics committee (Hospices Civils de Lyon) approved the study registered under number 20–156. This study was conducted in accordance with the Declaration of Helsinki.

### Treatment regimen

After being administered the initial loading dose, patients were treated with a Pro-Re-Nata (PRN) or Treat-and-Extend (TAE) regimen, at the discretion of the attending physician. For the PRN regimen, decisions for reinjection were based on the presence of neovascular activity assessed at each monthly visit [16]. For the TAE regimen, the treatment interval was reduced or extended by 2 weeks according to the presence or absence, respectively, of neovascular activity [6]. Switches between treatment regimens or drugs were allowed, at the discretion of the attending physician, and collected for review. In case of significant protocol violation, cases were excluded from analyses.

Neovascular activity was defined by one of the following criteria: decrease in best-corrected visual acuity (BCVA) ≥ 5 letters using the early treatment diabetic retinopathy study (ETDRS) chart, presence of a new retinal hemorrhage upon fundus ophthalmoscopy, presence or increase in IRF or SRF on SD-OCT, or dye leakage at fluorescein angiography (FA) [6, 16]. FA was used during follow-up to assess an evolution toward mixed type 1 and 2 MNV, suspected on SD-OCT. Such evolution excluded the eye from analyses.

### MNV classification and data collection

MNV subtype was determined according to the international consensus published by the CONAN group [8]. Type 1 MNV is defined by areas of neovascular complexes arising from the choroid and visualized as an elevation of the RPE by material with heterogeneous reflectivity on SD-OCT B scans; OCT-angiography (OCTA) shows vessels below the level of the RPE. FA shows stippled hyperfluorescence over an area of elevated RPE, which expands to coalesce in the later phases of the angiography. Late indocyanine green angiography (ICGA) finds staining of the lesion. As PCV can show an OCT pattern similar to that found in type 1 MNV, they were excluded in the absence of branching vascular network associated with aneurysmal dilatations on ICGA. In the presence of subretinal hyperreflective material, type 2 MNV components were excluded on the absence of focal discontinuity on SD-OCT, the absence of intrinsic flow into SHRM lesion on OCTA, and absence of early, well-defined, and intense hyperfluorescence on FA. Three retinal specialists (EG, KK, PG), masked to the interpretation of one another, reviewed all eyes for lesion subtyping based on multimodal imaging including the following mandatory tests: color fundus photography (Optos California, Optos PLC, Dunfermline, UK), FA, ICGA, and SD-OCT B scans (HRA Spectralis, Heidelberg Engineering, Heidelberg, Germany). OCT-angiography (OCTA – Angioplex, Carl Zeiss Meditec, Inc., Dublin, CA, US) was not mandatory but was analyzed if performed. In case of discrepancies between the reviews, a senior retinal specialist (LK or TM) evaluated the lesion type.

All data were collected retrospectively from the databases of the different centers by a retinal specialist (EG, KK, PG). At baseline, data collected included: gender, age, lens status, and history of hypertension. At each visit, data collected included: BCVA (ETDRS letters) and the presence of IRF, SRF, pigment epithelial detachment (PED), macular fibrosis, as well as macular atrophy on SD-OCT. Central macula thickness (CMT) was estimated using SD-OCT B scans on 1-mm thick ETDRS maps with the automated function of Spectralis software (Heidelberg eye explorer, Heidelberg Engineering, Heidelberg, Germany) and defined by the distance between the internal limiting membrane (ILM) and the RPE layer at the fovea. In case of error, manual corrections were performed. Any adverse event related to the intravitreal injection, or any event that could interfere with the treatment regimen or with the treatment outcomes, were also collected.

As previously described by the CONAN group [8], IRF was defined as cystoid spaces within the retina; SRF as a separation of the neurosensory retinal from the RPE by fluid; PED as an elevation of the RPE monolayer from the underlying Bruch’s membrane. Fibrosis was defined as a white or yellow-white accumulation of material on color fundus photography corresponding to a well-defined hyperreflective material in the subretinal space on SD-OCT, displaying blocked fluorescence of the underlying choroid in the FA early phase and hyperfluorescence due to tissue staining with no leakage in the late phase. Atrophy of the RPE was defined as the attenuation or the absence of RPE monolayer with overlying photoreceptor degeneration (i.e. thinning of the outer nuclear layer, loss of external limiting membrane, loss of ellipsoid zone and/or of interdigitation zone) and choroidal hypertransmission on SD-OCT irrespectively of size or localization from fovea. Fundus autofluorescence showing areas of hypoautofluorescence and near-infrared reflectance displaying hyperreflective areas with sharply demarcated borders were also analyzed to diagnose atrophy [8, 17, 18]. All these qualitative parameters were identified as “present” or “absent”.

Type 1 MNV develops under the RPE, thereby causing outer blood-retinal barrier disruption and SRF [19]. Secondarily, ELM disruption allows the presence of fluid in the neural retina, manifesting as IRF [5, 20]. To reflect the progression of fluid localization from subretinal compartment to intraretinal compartment allowed by ELM disruption, the study population was divided into 2 groups according to the fluid localization at baseline: the presence of SRF alone (SRF group), or presence of IRF associated or not with SRF (IRF ± SRF group).

### Outcomes

The primary outcome was the mean BCVA (ETDRS letters) at 12 and at 24 months, and the mean change in BCVA from baseline to 12 and from baseline to 24 months. BCVA at 12 or 24 months corresponded to the BCVA determined during the first visit at least 12 months and 24 months, respectively, after the baseline date. A subgroup analysis of the primary outcome according to the treatment regimen was performed to ensure comparability of eyes treated with PRN and TAE regimens.

Secondary outcomes were the CMT, the proportion of atrophy and fibrosis, and the total number of intravitreal injections. To allow comparisons with published studies, we also evaluated the specific effect of SRF and IRF presence at baseline on BCVA (ETDRS letters) over 24 months. We also investigated the impact of fluid fluctuation on the final BCVA (i.e. at 24 months) and on BCVA change from baseline. IRF recurrence was approximated by the proportion of visits between baseline and 24 months for which IRF was present.

### Statistical analyses

Data were expressed as mean with standard deviation (SD) for continuous variables and as count (percentage) for categorical variables. Comparisons between categorical variables were made using the Chi-squared test or Fisher’s exact test. Comparisons between continuous variables were tested using the non-parametric Kruskal-Wallis test. Linear regression analysis and the Kendall test were used to investigate the correlation between IRF fluctuation and BCVA outcomes. Variables correlated with the final BCVA and for which the p value was <0.05 in univariate analysis, as well as age, baseline BCVA, baseline CMT, treatment regimen, drug used, and numbers of intravitreal injections were included in a multivariate model. A p-value < 0.05 was considered statistically significant. All analyses were performed using R-3.5.3 software (R Foundation for Statistical Computing, Vienna, Austria).

## Results

### Patient characteristics

A total of 211 eyes from 177 patients were included. The mean (SD) age of patients was 77.7 (7.4) years, and 128 (60.7%) patients were female. At baseline, 146 (69.2%) eyes had SRF alone (these were therefore included in the SRF group), 11 (5.2%) had IRF alone, and 54 (25.6%) had a combination of IRF and SRF (these 65 eyes were therefore included in the IRF ± SRF group). SRF was present in 200 (94.8%) eyes, IRF in 65 (30.8%), and PED in all eyes. Baseline characteristics and demographics were well balanced according to fluid localization subgroup (Table 1) and between centers, except for treatment drug and regimen (Supplementary Table 1). The mean (SD) baseline BCVA was 66.2 (18.8) letters and was higher in the SRF group [70.2 (13.9) letters] than in the IRF ± SRF group [57.0 (24.6) letters; p < 0.001]. The mean (SD) CMT was 354.6 (104.7) µm and was higher in the IRF ± SRF group [403.1 (151.4) µm] than in the SRF group [333.1 (65.1) µm; p < 0.001]. A total of 9 (6.2%) eyes displayed fibrosis at baseline; fibrosis was present in 10.8% eyes of the IRF ± SRF group and 1.4% of the SRF group (p = 0.04). At baseline, atrophy was present in 25 (11.8%) eyes: 15.4% of the IRF ± SRF group and 10.3% of the SRF group (p = 0.29; Table 1).

**Table 1:** Baseline characteristics.

	Total cohort (N = 211)	SRF group (N = 146)	IRF ± SRF group (N = 65)
Age, years, mean (SD)	77.7 (7.4)	76.9 (7.3)	79.4 (7.15)
Female sex, n (%)	128 (60.7)	88 (60.3)	40 (61.5)
Right laterality, n (%)	100 (47.4)	65 (44.5)	35 (53.8)
Phakic status, n (%)	130 (61.6)	102 (69.9)	28 (43.1)
HBP, n (%)	89 (42.2)	63 (43.2)	26 (40)
Molecule used, n (%)			
Ranibizumab	88 (41.7)	53 (36.3)	35 (53.8)
Aflibercept	123 (58.3)	93 (63.7)	30 (46.2)
Treatment regimen, n (%)			
PRN	92 (43.8)	59 (40.4)	33 (51.6)
TAE	118 (56.2)	87 (59.6)	31 (48.4)
BCVA, ETDRS letters, mean (SD)	66.2 (18.8)	70.2 (13.9)	57 (24.6)
CMT, μm, mean (SD)	354.6 (104.7)	333.1 (65.1)	403.05 (151.4)
Presence of PED, n (%)	211 (100)	146 (100)	65 (100)
Presence of fibrosis, n (%)	9 (6.2)	2 (1.4)	7 (10.8)
Presence of atrophy, n (%)	25 (11.8)	15 (10.3)	10 (15.4)

*BCVA* best corrected visual acuity, *CMT* central macular thickness, *ETDRS* Early Treatment Diabetic Retinopathy Study, *HBP* high blood pressure, *IRF* intraretinal fluid, *PED* pigment epithelium detachment, *PRN* pro re nata, *SD* standard deviation, *SRF* subretinal fluid, *TAE* treat and extend.

### Functional and anatomical outcomes according to fluid localization at baseline

Data were available for 201 eyes (167 patients) at 12 months, and 157 eyes (130 patients) at 24 months. The baseline characteristics of eyes with available follow-up data at 24 months and those without were well balanced, except for the predominance of TAE amongst non-available-data eyes (Supplementary Table 2). At 12 months, the mean (SD) BCVA was 68.8 (20.0) letters and the mean (SD) BCVA gain from baseline was +2.8 (17.0) letters. The mean (SD) BCVA at 12 months was significantly higher in the SRF group [74.3 (12.6) letters] than in the IRF ± SRF group [56.9 (26.8) letters; p < 0.001], and the mean (SD) BCVA gain at 12 months from baseline was numerically higher in the SRF group [4.1 (14.3) letters] compared to the IRF ± SRF group [0.12 (21.5) letters], although the difference was not significant (p = 0.23). At 24 months, the mean (SD) BCVA was 67.9 (21.6) letters and the mean (SD) gain from baseline was +1.1 (18.7) letters. The mean (SD) BCVA at 24 months was significantly higher in the SRF group [72.2 (17.1) letters] than in the IRF ± SRF group [58.2 (27.3) letters; p < 0.001, Supplementary Fig. 1]. The mean (SD) BCVA gain at month 24 from baseline was numerically higher in the SRF group [+1.8 (17.4) letters] than in the IRF ± SRF group [−0.4 (21.5) letters], although the difference was not significant (p = 0.61, Table 2).

**Table 2:** Comparison of final BCVA, gain in BCVA, and CMT at month 12 and month 24 between groups.

		Total cohort	SRF group	IRF ± SRF group	p-value*
12 months	Number of eyes, n	201	137	64	
	BCVA, ETDRS letters, mean (SD)	68.8 (20.0)	74.3 (12.6)	56.9 (26.8)	p < 0.001
	Gain in BCVA, ETDRS letters, mean (SD)	2.8 (17.0)	4.1 (14.3)	0.12 (21.5)	0.23
	CMT, μm, mean (SD)	289.3 (74.9)	286.8 (60.6)	294.6 (99.2)	0.81
24 Months	Number of eyes, n	157	109	48	
	BCVA, ETDRS letters, mean (SD)	67.9 (21.6)	72.2 (17.1)	58.2 (27.3)	p < 0.001
	Gain in BCVA, ETDRS letters, mean (SD)	1.1 (18.7)	1.8 (17.4)	−0.4 (21.5)	0.61
	CMT, μm, mean (SD)	283.4 (73.6)	280.7 (68.6)	289.4 (84.5)	0.85

*BCVA* best-corrected visual acuity, *CMT* central macular thickness, *ETDRS* Early Treatment Diabetic Retinopathy Study, *IRF* intraretinal fluid, *SD* standard deviation *SRF* subretinal fluid.

*p-values: comparison between the SRF group and the IRF ± SRF group.

According to the treatment regimen, mean BCVA remained significantly higher in the SRF group than the IRF ± SRF group at 12 and 24 months for both TAE (p = 0.002 and p = 0.03, respectively) and PRN regimens (p < 0.001 and p = 0.01, respectively). Moreover, BCVA gain was significantly better for SRF group than IRF ± SRF only for eyes treated with TAE regimen at 24 months (p = 0.045), but not at 12 months (p = 0.08) and not significantly different at 12 and 24 months for eyes treated with PRN regimen (p = 0.9 and p = 0.14, respectively; Supplementary Tables 3 and 4).

**Fig. 1:**
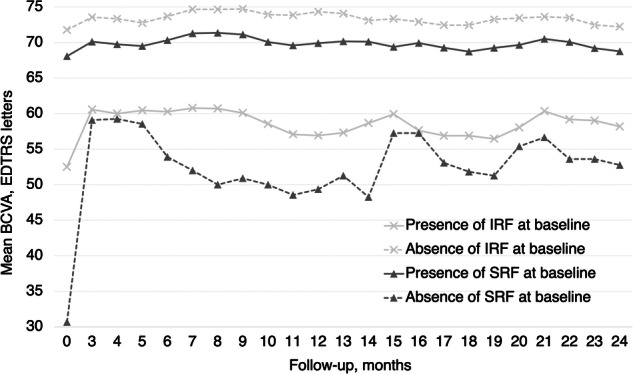
BCVA according to the presence of IRF and SRF from baseline to month 24. BCVA best-corrected visual acuity, ETDRS early treatment diabetic retinopathy study, IRF intraretinal fluid, SRF subretinal fluid.

According to the specific presence of fluid (independently of the previous group classification), the mean (SD) BCVA at 12 months was significantly lower among eyes presenting IRF at baseline [56.9 (26.8) letters] than among those that did not [74.3 (12.6) letters; p < 0.001], and significantly higher among eyes presenting SRF at baseline [69.9 (18.7) letters] than among those that did not [49.4 (31.3) letters; p = 0.02]. The mean (SD) BCVA at 24 months was significantly lower among eyes presenting IRF at baseline [58.2 (27.3) letters] than among those that did not [72.2 (17.1) letters; p < 0.001], but not significantly higher among eyes presenting SRF at baseline [68.8 (20.8) letters] than among those that did not [52.7 (32.1) letters; p = 0.13; Fig. 1]. The recurrence of IRF during follow-up was significantly but weakly correlated with lower BCVA (R^2^ = −0.18, p = 0.003).

The mean (SD) CMT was 289.3 (74.9) µm at 12 months and 283.4 (76.6) µm at 24 months. There was no significant difference in CMT between the SRF and the IRF ± SRF group at 12 months (p = 0.81) and at 24 months (p = 0.85; Table 2).

### Parameters associated with final BCVA

**Fig. 2:**
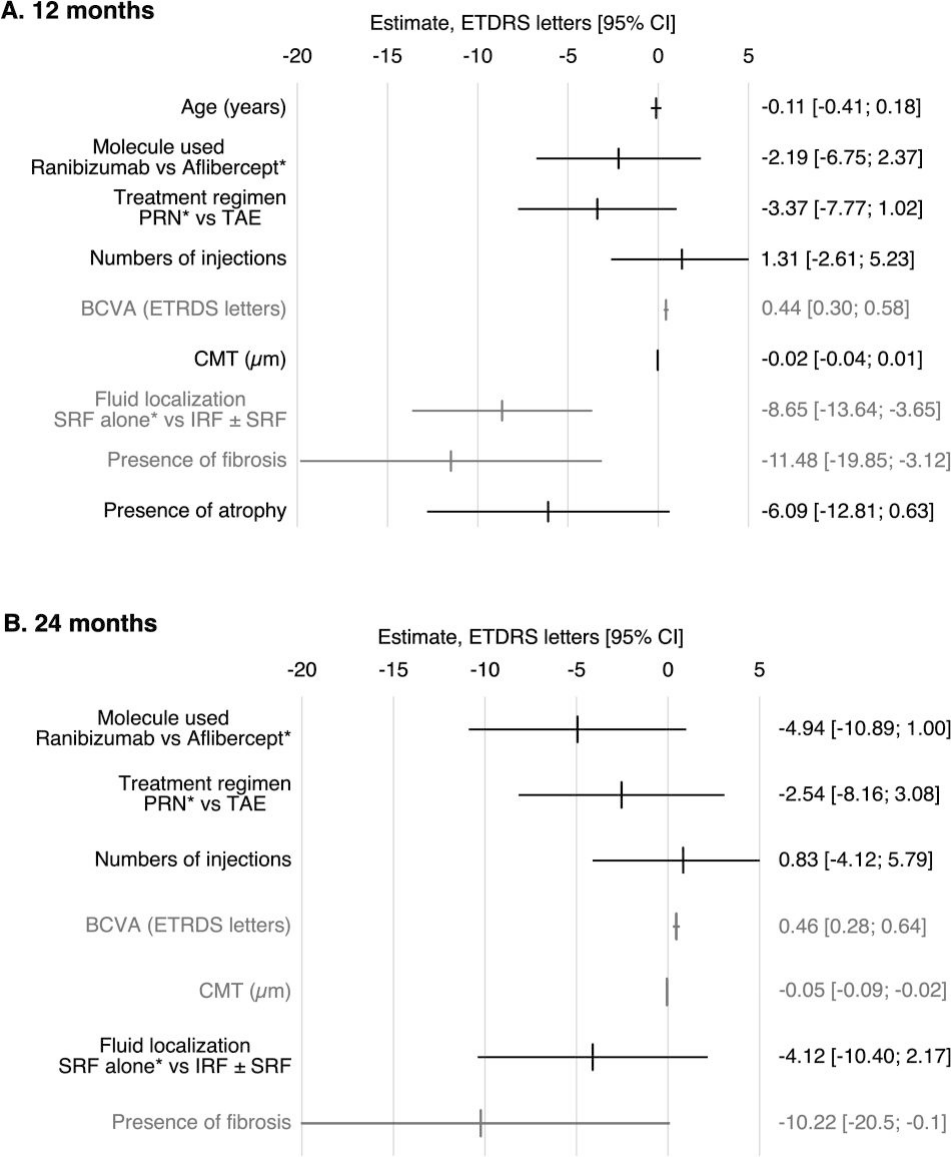
Association between baseline variables and final best corrected visual acuity (BCVA) at 12 and 24 months. The variables marked with an asterix are the reference variable. **A** Baseline BCVA, baseline fluid localization and baseline presence of fibrosis are significantly associated with BCVA at 12 months. **B** Baseline BCVA, the baseline CMT and baseline presence of fibrosis are significantly associated with BCVA at 24 months. BCVA best-corrected visual acuity; CI confidence interval; CMT central macular thickness; ETDRS Early Treatment Diabetic Retinopathy Study; IRF intraretinal fluid; PED pigment epithelium detachment; SRF subretinal fluid.

In the total cohort, age, baseline BCVA, baseline CMT, fluid localization, presence of fibrosis, and presence of atrophy were found to be significantly associated with BCVA at 12 months in univariate analysis. These were included in the multivariate analysis, and BCVA at 12 months was found to be significantly associated with baseline BCVA (estimate 0.44, 95% CI [0.30; 0.58], p < 0.001), fluid localization (IRF ± SRF group, estimate −8.65, 95% CI [−13.64; −3.65], p < 0.001), and presence of fibrosis at baseline (estimate −11.48, 95% CI [−19.85; −3.12], p = 0.007). Similarly, baseline BCVA, baseline CMT, fluid localization, and presence of fibrosis at baseline were found to be significantly associated with BCVA at 24 months in univariate analysis. These were included in the multivariate analysis, and BCVA at month 24 was found to be significantly associated with baseline BCVA (estimate 0.46, 95% CI [0.28; 0.64], p < 0.001), baseline CMT (estimate −0.05, 95% CI [−0.09; −0.02], p = 0.004), and presence of fibrosis at baseline (estimate −10.22, 95% CI [−20.53; −0.10], p = 0.049); the presence of IRF was not significantly associated with lower final BCVA (estimate −4.12, 95% CI [−10.40; 2.17], p = 0.19; Supplementary Table 5, Fig. 2).

### Analysis of fibrosis and atrophy

Fibrosis was present in 9/211 (6.2%) eyes at baseline, 19/201 (9.5%) at 12 months, and 14/157 (8.9%) at 24 months. In the SRF group, there was no significant difference between the frequency of fibrosis at baseline (2/146, 1.4%) and at 12 months (9/137, 6.6%; p = 0.99), nor between baseline and 24 months (6/109, 5.5%; p = 0.99). In the IRF ± SRF group, there was no significant difference between the frequency of fibrosis at baseline (7/65, 10.8%) and at 12 months (10/64, 15.6%; p = 0.33), nor between baseline and 24 months (8/48, 16.7%; p = 0.58). Fibrosis was significantly more frequent in the IRF ± SRF group than in the SRF group at 12 months (p = 0.04) and at 24 months (p = 0.03).

Atrophy was present in 25/211 (11.8%) eyes at baseline, 42/201 (20.9%) at 12 months, and 42/147 (26.8%) at 24 months. In the SRF group, there was no significant difference between the frequency of atrophy at baseline (15/146, 10.3%) and at 12 months (19/137, 13.9%; p = 0.22), nor between baseline and 24 months (20/109, 18.3%; p = 0.12). In the IRF ± SRF group, the frequency of atrophy was significantly lower at baseline (10/65, 15.4%) than at 12 months (23/64, 35.9%; p = 0.01), and at 24 months (22/48, 45.3%; p = 0.049). Atrophy was significantly less frequent in the SRF group than in the IRF ± SRF group at 12 months (p < 0.001) and at 24 months (p < 0.001, Supplementary Fig. 2).

### Intravitreal injections

In the total cohort, the mean (SD) number of intravitreal injections was 8.2 (2.7) at 12 months and 13.7 (5.0) at 24 months. There was no significant difference in the mean (SD) number of injections between the SRF and IRF ± SRF groups at 12 months [8.2 (2.9) versus 8.0 (2.5), respectively, p = 0.5], and at 24 months [13.8 (5.1) versus 13.3 (4.7), respectively, p = 0.43].

For the TAE regimen, the mean (SD) interval between the last two injections was 68.1 (76.3) days at 12 months, and 81.0 (79.7) days at 24 months. The mean (SD) total number of injections was 9.0 (2.4) at 12 months, and 14.6 (4.7) at 24 months. For the PRN regimen, the mean (SD) interval between the last two injections was 78.9 (82.2) days at 12 months, and 81.6 (86.9) days at 24 months. The mean (SD) total number of injections was 7.1 (2.8) at 12 months, and 12.8 (5.1) at 24 months.

There was no significant difference between the SRF and IRF ± SRF groups in terms of the numbers of injections at 12 and 24 months for TAE (p = 0.2 and p = 0.3, respectively) and PRN (p = 0.4 and p = 0.9, respectively) regimens, as well as for the interval between the last two injections at 12 and 24 months for TAE regimen (p = 0.21 and p = 0.12, respectively). For the PRN regimen, at 12 and 24 months, the mean (SD) the interval between the last two injections was significantly shorter in the SRF group than in the IRF ± SRF group at 12 months [69.74 (45.50) days vs 95.37 (122.91) days, p = 0.03] and at 24 months [71 (47.39) days vs 102.48 (133.39) days, p = 0.02].

## Discussion

The present study demonstrated that the presence of IRF at baseline in type 1 MNV was associated with lower BCVA at baseline and worse functional outcomes at both 12 and 24 months. The difference in the mean visual acuity according to the presence of IRF was 17.4 letters at 12 months and 14.0 letters at 24 months, illustrating the significant gap between eyes with and without IRF. The gain in BCVA was lower in case of IRF, but the difference with eyes with SRF alone did not reach significance, which may be explained in part by the ceiling effect as eyes with SRF only at baseline had higher initial BCVA and therefore had a lower potential to increase. Conversely, eyes with IRF at baseline had lower initial BCVA and reduced potential for further loss of vision. In addition, the smaller gains found in the present study that included only type 1 MNV could also probably be explained by the higher baseline BCVA of eyes diagnosed with nAMD and treated with anti-VEGF compared to other studies that included all MNV subtypes, especially randomized controlled trials [2, 16, 21]. The inclusion of only type 1 MNV herein may account for this discrepancy with previous studies, as it is well known that this neovascular type is associated with lower visual loss at baseline but also with better functional outcomes despite smaller BCVA gains [21, 22]. The absence of the effect of type 3 MNV may also increase differences between eyes with and without IRF at baseline as higher mean BCVA in type 3 MNV with IRF only at baseline has been reported [12, 23]. In the multivariate model presented herein, the presence of IRF at baseline was significantly associated with a loss of 8.65 letters at 12 months compared to eyes that did not. However, the loss of 4.12 letters at 24 months did not reach the significance, probably due to the loss of power between 12 and 24 months. Other factors significantly associated with BCVA at both 12 and 24 months were already described and include baseline BCVA [24] and the presence of fibrosis [25] at baseline. It is to note that treatment regimen was not significantly associated with BCVA at both 12 and 24 months in our multivariate model.

It has been widely reported that IRF is associated with lower visual outcomes in comparison to SRF. However, these studies included all patients with nAMD, regardless of the MNV subtype [9–11], and recent analyses of specific MNV subtypes questioned about the generalizability of these results. For instance, the recent study reported by Sharma et al. demonstrated that IRF-associated type 3 MNV had a better prognosis than SRF-associated type 3 MNV (i.e. BCVA difference of 15.3 letters) [12]. The authors hypothesized that in this MNV subtype specifically, IRF was associated with smaller lesions of lower stages (i.e. only intraretinal) and SRF appeared when the neovascular growth process reached the space under the RPE layer. Similarly, Sacconi et al. also demonstrated that the presence of baseline SRF was associated with poorer visual outcome for type 3 MNV (i.e. approximate BCVA difference of 17 letters) [23]. These studies raise questions on the prognostic dogma based on fluid localization and propose that the fluid-associated prognosis is due to MNV subtype, rather than to the simple fluid localization within the retina. Accordingly, we investigated the prognosis of fluid related to type 1 MNV, the most frequent MNV subtype encountered in nAMD, and therefore the most frequently included in randomized controlled trials and observational studies. The results for type 1 MNV presented herein are in line with large cohorts that included all MNV subtypes [9, 10], confirming that the results in such studies were mainly driven by type 1 MNV. Post-hoc analysis of randomized controlled trials found that the BCVA in MNV that displayed IRF at baseline was 8 to 8.2 letters lower than in MNV that did not display IRF at baseline at 12 months, and that this was 7 to 11 letters lower at 24 months [9, 10, 26]. Herein, the higher BCVA difference between eyes with and without IRF could be explained by the absence of the balancing effect related to type 3 MNV. To our knowledge, only one study reported by Kim et al. [15] has specifically analyzed type 1 MNV and found similar results to that reported in the present study, notably the presence of baseline IRF associated with lower BCVA and the presence of SRF associated with higher BCVA at 24 months, although their cohort was relatively small (79 eyes).

**Fig. 3:**
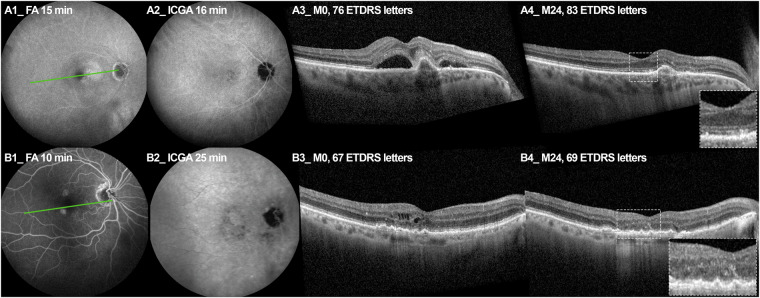
Panel of 2 cases displaying typical evolution with SRF alone and IRF + SRF from baseline to 24 months. **A** At baseline, FA displayed multiple drusen associated with ill-defined hyperfluorescence (A1) corresponding to late staining on ICGA (A2) and SRF opposite to a PED on OCT B scans (A3), defining a type 1 MNV. At month 24 (A4), ELM and EZ-IZ complex and RPE remained intact (dotted magnification). **B** At baseline, FA displayed ill-defined hyperfluorescence (B1) corresponding to a late staining on ICGA (B2) and IRF opposite to a PED on OCT-B scans (B3) defining a type 1 MNV. At 24 months (B4), OCT-B scans displayed ELM disruption, interruption of IZ-EZ complex with alteration of the RPE and hypersignal transmission to the choroid.

The results of the present study are consistent with the pathophysiology of type 1 MNV. This neovascular subtype, originating from the choroid, generally grows insidiously beneath the retina, between the Bruch’s membrane and the RPE layer, within a PED [14]. The development of the MNV under the RPE causes an alteration of the external blood-retinal barrier [19] that can lead to the accumulation of fluid in the subretinal space (i.e. SRF). Secondarily, an alteration of the ELM allows the presence of fluid within the retina layers, forming intraretinal cysts (i.e. IRF) [20]. As it has already been demonstrated that IRF was associated with more frequent alterations of ellipsoid zone and lower BCVA [27], this progression in fluid localization could be an explanation for the worse prognosis associated with IRF in type 1 MNV with more severe retinal alterations, especially on photoreceptors and RPE (Fig. 3) [5]. However, we found that about 95% of eyes presented SRF, almost a third presented IRF, and a quarter presented an association of both. Even though this proportion for SRF was above those described in a Korean cohort [15], it did not reach 100%. This could be due to the chronicity of the lesion that totally resorbed SRF by RPE pump, but not IRF.

We found a significant correlation of IRF recurrence with lower BCVA at 24 months, although it was weak. Chakravarthy et al. [28] included 403 eyes with nAMD and evaluated the impact of fluid fluctuations using the mean volume of fluid during the first 24 months; despite the absence of statistical tests, the recurrence of IRF seemed strongly associated with lower BCVA at 24 months whereas the recurrence of SRF was only weakly associated with lower BCVA. In addition, in a post-hoc analysis of the CATT study [29], Schmidt-Erfurth et al. evaluated the effect of fluid volumes in the central 1 mm of the macula on BCVA during a 24-month follow-up; IRF was significantly associated with a decrease of 4.0 letters per 0.1 mm^3^ and SRF with a gain of +1.1 letters per 0.1 mm^3^. It is also of note that persistent SRF in type 1 MNV during follow-up has been shown to be compatible with good BCVA, and also a non-significant difference in BCVA when compared to non-persistent SRF [30]. Taken together, these results indicate that IRF is related to a worse prognosis than SRF, and we suggest herein that this association is probably due to type 1 MNV.

The worse functional outcomes associated with IRF in type 1 MNV could be due to the presence of atrophy and/or fibrosis, although we found a relatively low prevalence of these complications at baseline, 11.8% and 6.2% respectively. These rates are in line with current literature [31, 32]. However, we showed herein that the presence of IRF at baseline was associated with worse outcomes, partially due to the increase in occurrence of atrophy and fibrosis during follow-up. We hypothesized that the presence of IRF could be due to alteration of outer retina and ELM, allowing the presence of fluid within the retina layers to form intraretinal cysts [20]. IRF in type 1 MNV could be associated with more severe retinal alterations, especially on photoreceptors and RPE, explaining atrophy and fibrosis. Similarly to other studies that have analyzed the presence of these complications in cohorts of nAMD irrespective of MNV subtype, herein these two complications were more frequent in the presence of IRF compared to SRF alone in type 1 MNV specifically. One should note that the overall frequency of atrophy in the present study was relatively high at the end of the follow-up (about a quarter of eyes) in comparison with other studies analyzing atrophy in type 1 MNV [31], but also that type 1 MNV is generally associated with less frequent atrophy and fibrosis than other MNV subtypes [31] (the neovascularization localizing beneath the RPE layer acting as a recapitulation of the choriocapillaris supplying the dysfunctional RPE [33]). However, the greater sensitivity for the detection of earlier stages of atrophy by the use of SD-OCT [17, 18] could also explain the higher frequency, as already reported elsewhere [31]. It should also be noted that the definition of atrophy irrespective of its localization from fovea may explain the absence of significant association with BCVA at 12 and 24 months in the multivariate model.

We acknowledge several limitations of the present study. The retrospective nature and the clinical decisions made by local physicians did not allow the visits to be at regular intervals as recommended for TAE (at precise extension timing) and PRN regimens (monthly follow-up) [6, 7]. Furthermore, the detection of fluid recurrence may have been delayed for some patients. To limit the impact of deviations from the protocol, each case was reviewed individually and was not included if a significant deviation was found. Nevertheless, the aim of this study was to explore the prognostic value of fluid in type 1 MNV and such deviations should not induce significant bias. In addition, the risk of MNV subtype misclassification was prevented by a triple reading of each baseline multimodal imaging, and in case of disagreement, by the review by a fourth senior evaluator. We used the CONAN group recommendations [8] to accurately classify MNV types. However, other secondary outcome measures such as fibrosis and atrophy were not assessed by masked investigators and could be misclassified. To limit bias in the reporting of these associated variables and to facilitate comparison with other studies, we also used consensus definition for classifying the presence of fibrosis [8] and atrophy [17, 18]. Furthermore, PCV was excluded because of their baseline characteristics and response to anti-VEGF therapy which may differ from type 1 MNV. Finally, other factors could also play a role in functional outcomes, such as baseline lesion size, hyperreflective foci and geographic atrophy stages, but were not collected in the present study. In the future, the use of artificial intelligence would help to determine specific biomarkers of vision loss in case of type 1 MNV.

In conclusion, the present study demonstrated the impact of baseline fluid localization on BCVA in patients with type 1 MNV secondary to nAMD and treated with anti-VEGF. IRF was associated with worse visual outcomes and increased risk of developing fibrosis and atrophy. These results highlight the need for further studies evaluating the effect of fluid localization at baseline and during follow-up according to the anatomical classification of the causative MNV.

## Summary

### What was known before

- Post-hoc analyses of randomized controlled trials have evaluated the effect of baseline fluid localization and found baseline SFR associated with favorable visual outcomes and baseline IRF with poorer vision.
- However, the main drawback of these studies is that they included all MNV subtypes, despite the different pathophysiology and prognosis of these.
- For instance, recent evidence has demonstrated an inverse correlation: IRF was found associated with better visual outcome in type 3 MNV, and SRF with worse prognosis.

### What this study adds

- As type 1 is the more frequent MNV type, and according to the physiopathology, we hypothesized that the effect of fluid localization in type 1 MNV may explain the results of the post-hoc analyses pooling all MNV subtypes.
- We report herein, the largest cohort of type 1 MNV defined according to anatomical classification. We identified that only IRF at baseline was associated with lower visual outcomes, and the presence of SFR alone was associated with more favorable visual outcome at both months 12 and 24.
- IRF was also associated with more frequent fibrosis and atrophy. In addition, the results suggest that the MNV subtype should be identified before analyzing fluid compartment and the resulting prognosis.

## Supplementary information

Supplemental Table 1

Supplemental Table 2

Supplemental Table 3

Supplemental Table 4

Supplemental Table 5

Supplemental Figure 1

Supplemental Figure 2

Supplementary Figure Caption
