# Supplementary material for: Effect of baseline fluid localization on visual acuity and prognosis in type 1 macular neovascularization treated with anti-VEGF
Source: Eye (Lond). 2024 Jul 31;38(16):3161–8. doi: 10.1038/s41433-024-03256-1 (PMC11543923; doi:10.1038/s41433-024-03256-1)
Supplement: Supplementary file 1 — Supplemental Table 1 [file 41433_2024_3256_MOESM1_ESM.docx]

**Supplemental table 1:** Baseline characteristics according to center.

|  | **Center 1** | **Center 2** | **Center 3** | **Center 4** | **Center 5** | **Center 6** | **Center 7** |
| --- | --- | --- | --- | --- | --- | --- | --- |
| Number of eyes, n (%) | 34 (16.1) | 30 (14.2) | 19 (9.1) | 49 (23.2) | 11 (5.2) | 43 (20.4) | 23 (10.9) |
| Age, years, mean (SD) | 77.4 (6.5) | 78.8 (6.4) | 76.6 (6.5) | 76.5 (6.5) | 78.7 (8.0) | 77.6 (8.5) | 79.8 (7.8) |
| Female sex, n (%) | 22 (64.7) | 21 (70.0) | 11 (57.9) | 27 (55.1) | 8 (72.7) | 28 (65.1) | 10 (43.5) |
| Right laterality, n (%) | 17 (50.0) | 17 (56.7) | 10 (56.7) | 21 (42.9) | 6 (54.5) | 22 (51.2) | 7 (30.4) |
| Phakic status, n (%) | 22 (64.7) | 15 (50.0) | 14 (73.7) | 32 (65.3) | 8 (72.7) | 25 (58.1) | 12 (52.2) |
| HBP, n (%) | 12 (35.3) | 13 (43.3) | 7 (36.8) | 21 (42.9) | 6 (54.5) | 18 (41.9) | 12 (52.2) |
| Molecule used, n (%) |  |  |  |  |  |  |  |
| Ranibizumab | 18 (52.9) | 14 (46.7) | 7 (36.8) | 5 (10.2) | 5 (45.5) | 29 (67.4) | 8 (34.8) |
| Aflibercept | 16 (47.1) | 16 (53.3) | 12 (63.2) | 44 (89.8) | 6 (54.5) | 14 (32.6) | 15 (65.2) |
| Treatment regimen, n (%) |  |  |  |  |  |  |  |
| PRN | 24 (70.6) | 18 (60) | 1 (5.3) | 5 (10.2) | 10 (90.9) | 19 (45.2) | 13 (5.5) |
| TAE | 16 (47.1) | 16 (53.3) | 12 (63.2) | 44 (89.8) | 6 (54.5) | 14 (32.6) | 10 (43.5) |
| BCVA, ETDRS letters, mean (SD) | 66.7 (17.2) | 63.2 (22.5) | 65.9 (15.6) | 65.8 (15.4) | 70 (14.2) | 68.6 (22.3) | 56.9 (26.5) |
| CMT, μm, mean (SD) | 389.2 (155.2) | 338.8 (69.4) | 337.8 (67.3) | 338 (66) | 338.1 (68.5) | 350.8 (87.4) | 397.3 (156.2) |
| Presence of fluid at baseline, n (%)  SRF  IRF±SRF | 17 (50)  17 (50) | 19 (63.3)  11 (36.7) | 17 (89.5)  2 (10.5) | 41 (83.7)  8 (16.3) | 7 (63.6)  4 (36.4) | 29 (67.4)  14 (32.6) | 15 (65.2)  8 (34.8) |
| Presence of fibrosis, n (%) | 0 (0.0) | 2 (6.7) | 2 (10.5) | 9 (18.4) | 1 (9.1) | 0 (0.0) | 2 (8.7) |
| Presence of atrophy, n (%) | 2 (5.9) | 2 (6.7) | 5 (26.3) | 2 (4.1) | 1 (9.1) | 9 (20.9) | 3 (13.0) |

BCVA: best corrected visual acuity; CMT: central macular thickness; ETDRS: Early Treatment Diabetic Retinopathy Study; HBP: high blood pressure; IRF: intraretinal fluid; PRN: pro re nata; SD: standard deviation; SRF: subretinal fluid; TAE: treat and extend.

Summary text: This table display baseline characteristics according to the center. Baseline characteristics were balanced between groups except for treatment regimen and molecule used.
