# Supplementary material for: Effect of baseline fluid localization on visual acuity and prognosis in type 1 macular neovascularization treated with anti-VEGF
Source: Eye (Lond). 2024 Jul 31;38(16):3161–8. doi: 10.1038/s41433-024-03256-1 (PMC11543923; doi:10.1038/s41433-024-03256-1)
Supplement: Supplementary file 2 — Supplemental Table 2 [file 41433_2024_3256_MOESM2_ESM.docx]

**Supplemental table 2:** Baseline characteristics of eyes according to the availability of month-24 data.

|  | With 24-month data | Without 24-month data |
| --- | --- | --- |
| Number of eyes, n (%) | 157 (74.4) | 54 (25.6) |
| Age, years, mean (SD) | 77.7 (7.5) | 77.9 (7.0) |
| Female sex, n (%) | 97 (61.8) | 31 (57.4) |
| Right laterality, n (%) | 72 (45.9) | 28 (51.9) |
| Phakic status, n (%) | 96 (61.1) | 34 (63.0) |
| HBP, n (%) | 65 (41.4) | 24 (44.4) |
| Molecule used, n (%) |  |  |
| Ranibizumab | 70 (44.6) | 18 (33.3) |
| Aflibercept | 87 (55.4) | 36 (66.7) |
| Treatment regimen, n (%) |  |  |
| PRN | 82 (52.6) | 10 (18.5) |
| TAE | 74 (47.4) | 44 (81.5) |
| BCVA, ETDRS letters, mean (SD) | 66.8 (18.8) | 64.3 (19) |
| CMT, μm, mean (SD) | 344.2 (89.6) | 384.8 (136.1) |
| Presence of IRF, n (%) | 48 (30.6) | 17 (31.5) |
| Presence of SRF, n (%) | 149 (94.9) | 51 (94.4) |
| Presence of PED, n (%) | 157 (100) | 54 (100) |
| Presence of fibrosis, n (%) | 14 (8.9) | 2 (3.7) |
| Presence of atrophy, n (%) | 17 (10.8) | 8 (14.8) |

BCVA: best corrected visual acuity; CMT: central macular thickness; ETDRS: Early Treatment Diabetic Retinopathy Study; HBP: high blood pressure; IRF: intraretinal fluid; PED: pigment epithelium detachment; PRN: pro re nata; SD: standard deviation; SRF: subretinal fluid; TAE: treat and extend.

Summary text:This table display baseline characteristics according to the availability of month-24 data. From 211 eyes at baseline, 3 quarters were available at 24 months. All characteristics were balanced between groups except treatment regimen with higher proportion of TAE regiment amongst non-available-data eyes.
