# Supplementary material for: Effect of baseline fluid localization on visual acuity and prognosis in type 1 macular neovascularization treated with anti-VEGF
Source: Eye (Lond). 2024 Jul 31;38(16):3161–8. doi: 10.1038/s41433-024-03256-1 (PMC11543923; doi:10.1038/s41433-024-03256-1)
Supplement: Supplementary file 3 — Supplemental Table 3 [file 41433_2024_3256_MOESM3_ESM.docx]

**Supplemental Table 3:** Comparison of final BCVA, gain in BCVA, and CMT at month 12 and month 24 between groups for eyes treated in TAE.

|  | | Total cohort | SRF group | IRF±SRF group | p-value* |
| --- | --- | --- | --- | --- | --- |
| 12 months | Number of eyes, n | 110 | 79 | 31 |  |
|  | BCVA, ETDRS letters, mean (SD) | 66.7 (22.3) | 73.0 (12.7) | 50.6 (31.8) | 0.002 |
|  | Gain in BCVA, ETDRS letters, mean (SD) | 2.0 (16.6) | 4.8 (13.2) | -4.9 (21.8) | 0.081 |
|  | CMT, μm, mean (SD) | 292.5 (77.4) | 293.5 (62.2) | 289.8 (108) | 0.64 |
| 24 Months | Number of eyes, n | 74 | 54 | 20 |  |
|  | BCVA, ETDRS letters, mean (SD) | 66.6 (24.3) | 71.8 (17.5) | 52.5 (33.6) | 0.03 |
|  | Gain in BCVA, ETDRS letters, mean (SD) | 1.1 (3.8) | 3.8 (15.8) | -6.0 (21.0) | 0.045 |
|  | CMT, μm, mean (SD) | 276.0 (72.5) | 276.0 (62.0) | 276.3 (97.1) | 0.44 |

*p-values: comparison between the SRF group and the IRF±SRF group.

BCVA: best-corrected visual acuity; CMT: central macular thickness; ETDRS: Early Treatment Diabetic Retinopathy Study; IRF: intraretinal fluid; SD: standard deviation, SRF: subretinal fluid; TAE: treat and extend.

Summary text: This table display subgroup analysis of patients treated with a TAE regimen. BCVA was significantly higher in the SRF group than IRF ± SRF group at 12 and 24 months. BCVA gain was numerically higher in SRF group at 12 and 24 months but reach significance only at 24 months.
