# Supplementary material for: Effect of baseline fluid localization on visual acuity and prognosis in type 1 macular neovascularization treated with anti-VEGF
Source: Eye (Lond). 2024 Jul 31;38(16):3161–8. doi: 10.1038/s41433-024-03256-1 (PMC11543923; doi:10.1038/s41433-024-03256-1)
Supplement: Supplementary file 4 — Supplemental Table 4 [file 41433_2024_3256_MOESM4_ESM.docx]

**Supplemental Table 4:** Comparison of final BCVA, gain in BCVA, and CMT at month 12 and month 24 between groups for eyes treated in PRN.

|  | | Total cohort | SRF group | IRF±SRF group | p-value* |
| --- | --- | --- | --- | --- | --- |
| 12 months | Number of eyes, n | 90 | 58 | 32 |  |
|  | BCVA, ETDRS letters, mean (SD) | 71.2 (16.7) | 76.1 (12.5) | 62.3 (19.8) | <0.001 |
|  | Gain in BCVA, ETDRS letters, mean (SD) | 3.9 (17.6) | 3.2 (15.8) | 5.2 (20.6) | 0.9 |
|  | CMT, μm, mean (SD) | 285.9 (72.4) | 277.7 (57.6) | 300.7 (92.6) | 0.72 |
| 24 Months | Number of eyes, n | 82 | 55 | 27 |  |
|  | BCVA, ETDRS letters, mean (SD) | 69.3 (19) | 72.7 (16.8) | 62.6 (21.7) | 0.01 |
|  | Gain in BCVA, ETDRS letters, mean (SD) | 1.5 (19.4) | -0.1 (18.7) | 4.8 (20.7) | 0.14 |
|  | CMT, μm, mean (SD) | 290.4 (74.8) | 285.4 (74.7) | 300.6 (75.5) | 0.64 |

*p-values: comparison between the SRF group and the IRF±SRF group.

BCVA: best-corrected visual acuity; CMT: central macular thickness; ETDRS: Early Treatment Diabetic Retinopathy Study; IRF: intraretinal fluid; PRN: Pro Re Nata; SD: standard deviation, SRF: subretinal fluid.

Summary text: This table display subgroup analysis of patients treated with a PRN regimen. BCVA was significantly higher in the SRF group than IRF ± SRF group at 12 and 24 months. BCVA gain was not significantly different between SRF and IRF ± SRF group.
