# Supplementary material for: Effect of baseline fluid localization on visual acuity and prognosis in type 1 macular neovascularization treated with anti-VEGF
Source: Eye (Lond). 2024 Jul 31;38(16):3161–8. doi: 10.1038/s41433-024-03256-1 (PMC11543923; doi:10.1038/s41433-024-03256-1)
Supplement: Supplementary file 5 — Supplemental Table 5 [file 41433_2024_3256_MOESM5_ESM.docx]

**Supplemental Table 5:** Association between baseline variables and final best corrected visual acuity at 12 months (Table 5A) and 24 months (Table 5B).

| **A. 12 months** | Univariate analysis | | Multivariate analysis | |
| --- | --- | --- | --- | --- |
|  | Estimate [95% CI] | P-value | Estimate [95% CI] | P-value |
| Age (years) | -0.39 [-0.78; -0.01] | 0.04 | -0.11 [-0.41; 0.18] | 0.44 |
| Female sex | -3.59 [-9,33; 2.15] | 0.21 | - | - |
| Right laterality | -1.06 [-5.06; 2.94] | 0.60 | - | - |
| Phakic status | 2.85 [-2.96; 8.66] | 0.33 | - | - |
| HBP | -3.72 [-9.44; 2.00] | 0.19 | - | - |
| Drug used: |  |  |  |  |
| Ranibizumab | -1.47 [-7.19; 4.25] | 0.61 | -2.19 [-6.75; 2.37] | 0.34 |
| Aflibercept^†^ | - | - | - | - |
| Treatment regimen: |  |  |  |  |
| PRN^†^ | - | - | - | - |
| TAE | -4.53 [-10.21; 1.15] | 0.11 | -3.37 [-7.77; 1.02] | 0.13 |
| Numbers of IVT | 0.94 [-4.20; 6.07] | 0,72 | 1.31 [-2.61; 5.23] | 0.50 |
| BCVA (ETDRS letters) | 0.65 [0.54; 0.77] | < 0.001 | 0.44 [0.30; 0.58] | < 0.001 |
| CMT (μm) | -0.08 [-0.10; -0.05] | < 0.001 | -0.02 [-0.04; 0.01] | 0.08 |
| Fluid localization:  SRF alone^†^  IRF±SRF | -  -  -17.42 [-23.0; -11.9] | -  -  < 0.001 | -  -  -8.65 [-13.64; -3.65] | -  -  < 0.001 |
| Presence of fibrosis | -28.64 [-38.3; -19.0] | < 0.001 | -11.48 [-19.85; -3.12] | 0.007 |
| Presence of atrophy | -17.42 [-23.0; -11.9] | < 0.001 | -6.09 [-12.81; 0.63] | 0.07 |

| **B. 24 months** | Univariate model | | Multivariate model | |
| --- | --- | --- | --- | --- |
|  | Estimate [95% CI] | P-value | Estimate [95% CI] | P-value |
| Age (years) | -0.41 [-0.88; 0.05] | 0.07 | - | - |
| Female sex | -6.03 [-13.09; 1.03] | 0.09 | - | - |
| Right laterality | -3.83 [-8.70; 1.05] | 0.12 | - | - |
| Phakic status | 1.46 [-5.64; 8.56] | 0.68 | - | - |
| HBP | -2.11 [-9.13; 4.91] | 0.55 | - | - |
| Drug used: |  |  |  |  |
| Ranibizumab | -6.29 [-13.18; 0.60] | 0.07 | -4.94 [-10.89; 1.00] | 0.10 |
| Aflibercept^†^ | - | - | - | - |
| Treatment regimen: |  |  |  |  |
| PRN^†^ | - | - | - | - |
| TAE | -2.79 [-9.74; 4.17] | 0.42 | -2.54 [-8.16; 3.08] | 0.37 |
| Numbers of IVT | -0.87 [-6.92; 5.18] | 0.77 | 0.83 [-4.12; 5.79] | 0.74 |
| BCVA (ETDRS letters) | 0.67 [0.52; 0.82] | <0.001 | 0.46 [0.28; 0.64] | <0.001 |
| CMT (μm) | -0.11 [-0.15; -0.08] | <0.001 | -0.05 [-0.09; -0.02] | 0.004 |
| Fluid localization:  SRF alone^†^  IRF±SRF | -  -14.05 [-21.2; -6.9] | -  <0.001 | -  -4.12 [-10.40; 2.17] | -  0.19 |
| Presence of fibrosis | -27.39 [-38.7; -16.1] | <0.001 | -10.22 [-20.53; -0.10] | 0.045 |
| Presence of atrophy | -8.64 [-19.7; 2.4] | 0.12 | - | - |

*Variables for which the p-value was <0.05 in univariate analysis were included in the multivariate analysis.

†Reference

BCVA: best corrected visual acuity; CI: confidence interval; CMT: central macular thickness; ETDRS: Early Treatment Diabetic Retinopathy Study; HBP: high blood pressure; IRF: intraretinal fluid; IVT: intravitreal injection; PED: pigment epithelium detachment; PRN: pro re nata; RE: right eye; SRF: subretinal fluid; TAE: treat and extend.

Summary text: This table display univariate and multivariate models at 12 and 24 months. In the multivariate model, baseline BCVA and presence of fibrosis at baseline were significantly associated with BCVA at both 12 and 24 months; fluid localization was only associated with BCVA at 12 months but not 24 months.
