# Supplementary figures and images for: Effect of baseline fluid localization on visual acuity and prognosis in type 1 macular neovascularization treated with anti-VEGF
Source: Eye (Lond). 2024 Jul 31;38(16):3161–8. doi: 10.1038/s41433-024-03256-1 (PMC11543923; doi:10.1038/s41433-024-03256-1)

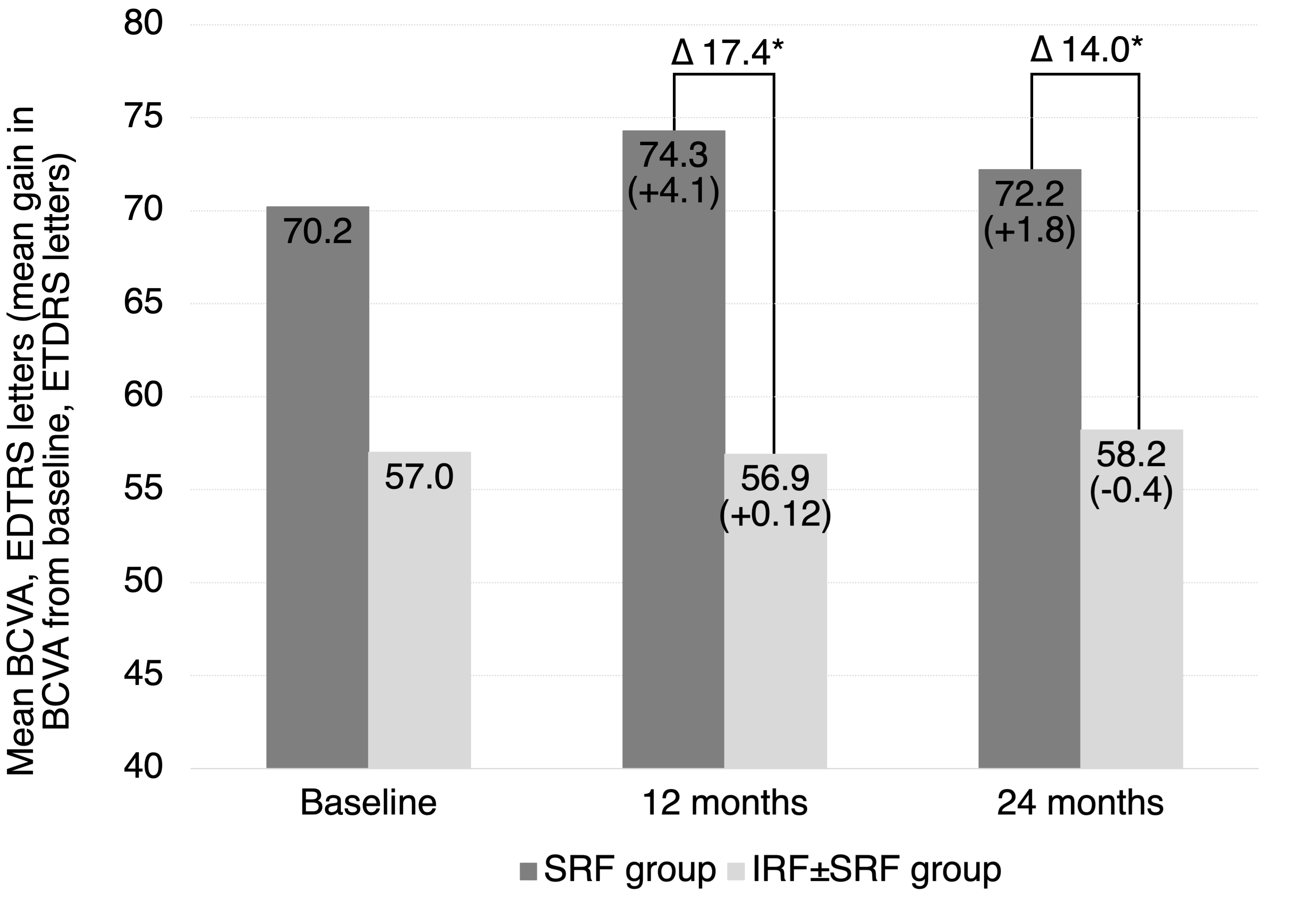

Supplement: Supplementary file 6 — Supplemental Figure 1 [file 41433_2024_3256_MOESM6_ESM.tif]

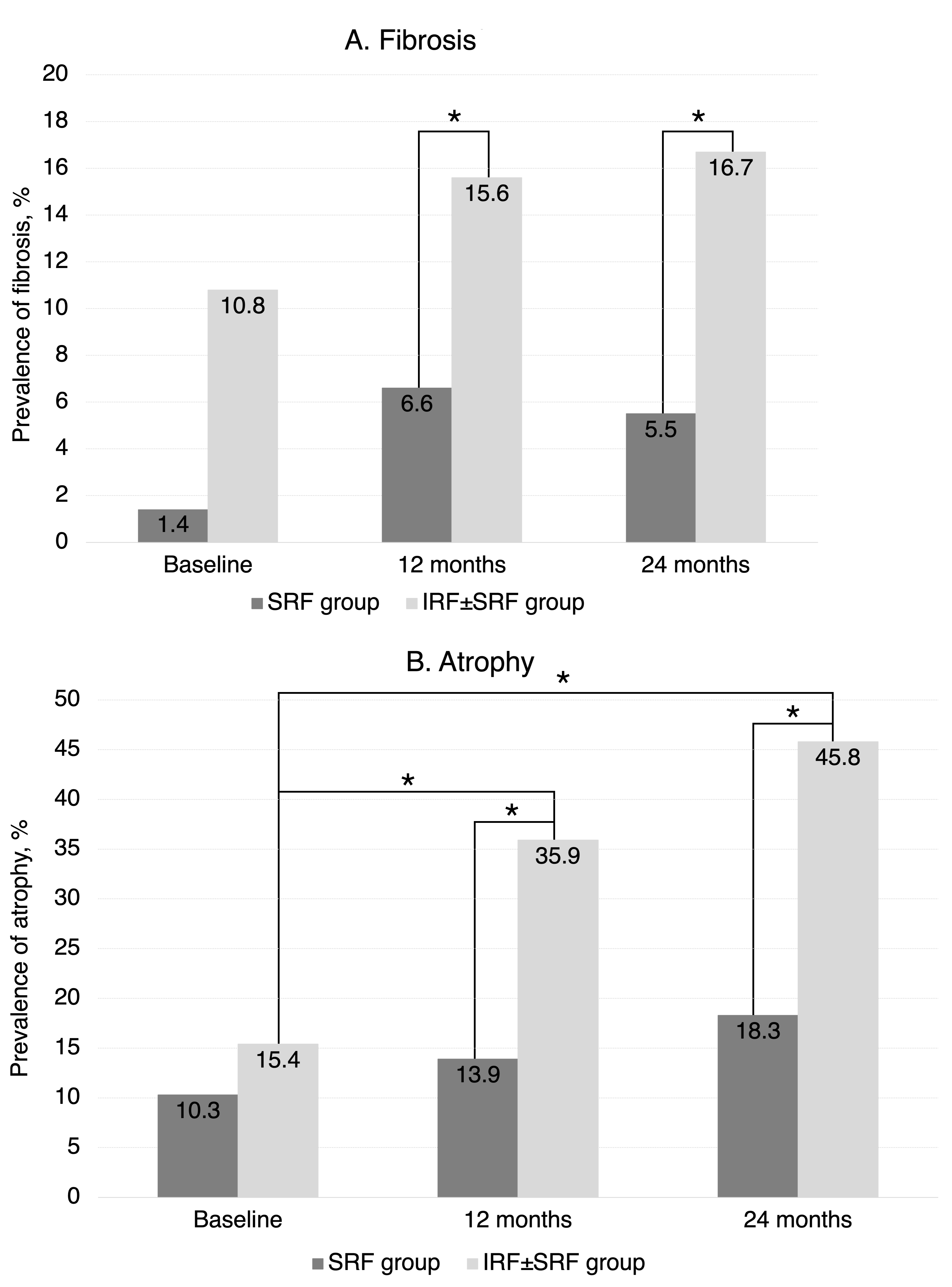

Supplement: Supplementary file 7 — Supplemental Figure 2 [file 41433_2024_3256_MOESM7_ESM.tif]
