# Supplementary material for: Effect of baseline fluid localization on visual acuity and prognosis in type 1 macular neovascularization treated with anti-VEGF
Source: Eye (Lond). 2024 Jul 31;38(16):3161–8. doi: 10.1038/s41433-024-03256-1 (PMC11543923; doi:10.1038/s41433-024-03256-1)
Supplement: Supplementary file 8 — Supplementary Figure Caption [file 41433_2024_3256_MOESM8_ESM.docx]

**Supplemental figure 1:** Mean BCVA at baseline, at 12 months and at 24 months according to fluid localization.

*p-value < 0.05.

BCVA: best-corrected visual acuity; ETDRS: early treatment diabetic retinopathy study; IRF: intraretinal fluid; SRF: subretinal fluid.

**Supplemental figure 2:** Development of fibrosis (A) and atrophy (B) at month 12 and month 24 according to the presence of intraretinal and subretinal fluid at baseline.

*p-values < 0.05.

IRF: intraretinal fluid; SRF: subretinal fluid
